# Preoperative Antibiotic Prophylaxis in Elective Minor Surgical Procedures among Adults in Southern Italy

**DOI:** 10.3390/antibiotics9100713

**Published:** 2020-10-18

**Authors:** Giorgia Della Polla, Aida Bianco, Silvia Mazzea, Francesco Napolitano, Italo Francesco Angelillo

**Affiliations:** 1 Department of Experimental Medicine, University of Campania “Luigi Vanvitelli”, Via Luciano Armanni, 5 80138 Naples, Italy; giorgia.dellapolla@unicampania.it (G.D.P.); francesco.napolitano2@unicampania.it (F.N.); 2 Department of Health Sciences, University “Magna Graecia” of Catanzaro, Via Tommaso Campanella, 115 88100 Catanzaro, Italy; a.bianco@unicz.it (A.B.); silvia.mazzea@studenti.unicz.it (S.M.)

**Keywords:** appropriateness, minor surgical procedures, preoperative antibiotic prophylaxis, Southern Italy

## Abstract

Little is known regarding the factors associated with surgical antibiotic prophylaxis (SAP) compliance in elective minor surgery. The purposes of this cross-sectional study were to identify the frequency of inappropriate SAP administration and to understand the characteristics associated with such inappropriateness in a sample of elective minor surgical procedures. The study was performed between May and July 2019 among a random sample of patients aged 18 years and older in seven public hospitals randomly selected in the Campania and Calabria Regions of Italy. Globally, only 45% of SAP approaches were deemed completely in accordance with the evidence-based guidelines. Patients with an ordinary admission, those who underwent local anesthesia, those receiving plastic and reconstructive and ophthalmology surgery, and those who had not received a prosthetic implant were more likely to receive an appropriate SAP approach; those receiving obstetrics, gynecological, and urological surgical procedures were less likely than those who underwent abdominal, vascular, and breast surgery. The course of antibiotic prophylaxis was not consistent with the guidelines in 48.5% procedures with one or more reasons for inappropriateness. Appropriate time of the SAP administration was more frequently observed in patients who were older, those with a Charlson comorbidity index of 0, those who did not receive a prosthetic implant, and those receiving plastic and reconstructive surgery; it was less likely in patients receiving obstetrics, gynecological, and urological surgeries compared with those who underwent abdominal, vascular, and breast surgery. Aspects of SAP that need to be improved are molecule choice, time of administration, and specific surgical procedures. Hospital managers should involve surgeons and anesthesiologists in initiatives tailored to optimize SAP prescribing.

## 1. Introduction

Surgical site infections (SSIs) are among the most common healthcare-associated infections (HAI) and are associated with longer post-operative hospital stays, additional surgical procedures, treatment in an intensive care unit, and higher mortality [1]. Consequently, surgical antibiotic prophylaxis (SAP) has to be implemented prior to a procedure for the prevention of these infections at the surgical site [2]. Indeed, it has been demonstrated that its adequate use can reduce the incidence of SSI in up to 50% of cases [3,4], whereas an unnecessary antibiotic use increases the selective pressure favoring the emergence of antibiotic resistance (AR) and represents an important target for antimicrobial stewardship efforts in hospital settings [5,6,7]. Guidelines on the administration of SAP have been published [8,9] and indication, molecule choice, timing, and duration are essential components of hospital-based antimicrobial stewardship programs. However, previous studies have shown wide variation in compliance [10,11,12,13,14] with nonoptimal adherence with overuse in those interventions where there is no indication or underuse when indicated, and inappropriate use of third-generation cephalosporins [15]. Therefore, it is crucial to determine the occurrence of an indication, to select the appropriate agent that covers the likely pathogens on wound contamination, and to administer sufficient bactericidal concentrations for the whole period during which the incision is open and at risk of contamination [7].

Despite several studies focused on antibiotic prescribing habits in conjunction with surgeries in different countries having been recently published [16,17,18], little is known regarding the factors associated with SAP compliance in elective minor surgery [19,20,21]. Consequently, it is imperative to acquire such information. Therefore, the purposes of the present study were to identify the frequency of unnecessary and inappropriate SAP administration and to understand the characteristics associated with such inappropriateness in a sample of elective minor surgical procedures in Italian hospitals.

## 2. Results

All 602 randomly selected patients who underwent elective minor surgery during the study period agreed to participate. More than half were females, the mean age was 61.7 years, 66.8% had at least one chronic disease, 10.3% self-reported the use of antibiotics in the previous month, 72.8% were admitted in surgical specialty wards, 62.1% had an ordinary admission, 79.4% of the procedures were clean, the mean duration of surgery was 23 min, and the majority of the interventions was under local anesthesia (Table 1).

Among the 602 patients included in the study, 488 (81.1%) received SAP. SAP was administered in 322 (97.3%) of the procedures in which it was indicated, and it was not administered in 105 (38.8%) of those in which it was not indicated. Globally, 271 (45%) SAP approaches were deemed completely in accordance with the evidence-based guidelines. Among the 331 patients eligible for the prophylaxis, only 2.7% of them did not receive antibiotics, whereas among the 271 procedures for which the SAP was not indicated, almost two-thirds (61.3%) received antibiotics.

SAP administration in the 322 procedures with indication according to drug choice, route of administration, timing, duration, and dosage are presented in Table 2.

The course of antibiotic prophylaxis was not consistent with the guidelines in 156 (48.5%) procedures with one or more reasons for inappropriateness. The reasons for the inappropriate administration were incorrect timing (39.9%), use of a broad-spectrum molecule (18.6%), duration (4.7%), route (4.7%), and dosage (4.4%). The most frequently inappropriately used agents were ceftriaxone (60.8%), cefuroxime (42.3%), cefazoline (11.6%), and levofloxacine (7.5%).

The SAP approach according to the various characteristics of patients and surgical procedures is illustrated in Table 1. Bivariate analysis showed that the patients that had received SAP completely in accordance with the evidence-based guidelines were more likely to be older (χ^2^ = −9.43; 597 df; *p* < 0.001), female (χ^2^ = 10.48; 1 df; *p* = 0.001), with a Charlson index ≥ 1 (χ^2^ = 18.39; 1 df; *p* < 0.001), with procedures performed under local anesthesia (χ^2^ = 19.73; 1 df; *p* < 0.001), in clean surgical wounds (χ^2^ = 22.84; 2 df; *p* < 0.001), and in those without a prosthetic implant (χ^2^ = 71.7; 1 df; *p* < 0.001). The results of the multivariate stepwise logistic regression analysis are showed in Table 3.

Patients with an ordinary admission (OR = 2.93; CI 95% 1.52–5.66), those who underwent local anesthesia (OR = 8.45; CI 95% 3.13–22.85), those receiving plastic and reconstructive (OR = 6.14; CI 95% 1.65–22.83) and ophthalmology (OR = 2.56; CI 95% 1.4–4.72) surgery, and those who had not received a prosthetic implant (OR = 0.01; CI 95% 0.01–0.02) were more likely to receive an appropriate SAP approach. However, obstetrics, gynecological, and urological surgical procedures (OR = 0.14; CI 95% 0.03–0.65) were less likely to receive the SAP approach completely in accordance with the evidence-based guidelines compared with those who underwent abdominal, vascular, and breast surgery (Model 1).

Out of all 488 patients who received SAP, 287 (58.8%) had an appropriate timing of administration of the antibiotic. At the bivariate analysis, as illustrated in Table 1, the appropriate timing of SAP administration was significantly more likely in patients admitted in surgical specialty wards (χ^2^ = 63.35; 1 df; *p* < 0.001), who had not received a prosthetic implant (χ^2^ = 91.36; 1 df; *p* < 0.001), and with a lower duration of surgical intervention (τ = 5.98; 544 df; *p* < 0.001). The results of the multivariate logistic regression analysis showed that the appropriate time of the SAP administration was more frequently observed in patients who were older (OR = 1.03; CI 95% 1.01–1.05), who did not receive a prosthetic implant (OR = 0.06; CI 95% 0.03–0.11), who had a Charlson comorbidity index of 0 (OR = 0.22; CI 95% 0.11–0.48), and those receiving plastic and reconstructive surgery (OR = 20.33; CI 95% 2.11–195.9), whereas it was less likely in patients receiving obstetrics, gynecological, and urological surgeries (OR = 0.31; CI 95% 0.13–0.71) compared with those who underwent abdominal, vascular, and breast surgery (Model 2 in Table 3).

## 3. Discussion

The study represents one of the few attempts in the literature to evaluate the approach of prescription of SAP in elective minor surgical procedures according to the current guidelines and the factors influencing such adherence in Italy. This could be used to design and disseminate specific measures to prevent SSIs in this type of operation, since it is obvious that without understanding physicians’ practice regarding SAP, any educational interventions will lead to failure of these initiatives. This survey has three key findings.

First, a gap between scientific evidence and clinical practice has been observed. Indeed, the results showed that the implementation of best surgical practices failed, with surgeons being prone to prescribe an antibiotic for procedures when it is not indicated (61.3%) than to underuse, negating SAP when it is indicated (2.7%). This practice underlined that patients not receiving SAP should be considered a low-priority target for SAP optimization, and surgeons are more concerned about the risk of SSIs than by the risks related to antibiotic misuse, such as the emergence of resistant microorganisms or side effects. These results may be explained by the fact that surgeons mistakenly believe that the overuse of antibiotics and extending SAP could reduce the risk of SSIs.

Second, significant gaps in adherence to current evidence-based recommendations have also been found when SAP administration is indicated. The overall compliance rate, such as appropriate molecule, route of administration, timing, duration, and dosage, was unsatisfactory (48.5%). Huge variations were observed with values of appropriate SAP ranging from 0.3% to 84.5% [14]. The results of the multivariate analysis showed that SAP was more likely to be appropriately administered in ordinary admissions compared with day-surgery. In the field of minor surgery, most patients are discharged on the same day after surgery, and surgeons have difficulty in monitoring the post-operative status of the surgical wound. As a result, SAP is inappropriately administered in the hope of preventing SSIs. Other determinants of inappropriateness include the interventions under general anesthesia and with prosthetic implants. Regarding anesthesia, although not conclusive, evidence has suggested that in selected surgical procedures, local compared with general may reduce the risk of SSI throughout an improvement of tissue oxygenation [22,23], and this could likely avoid SAP overuse.

A thorough analysis of the ways SAP was administered according to each of the single components shows a very concerning scenario. The main components that contributed to the low overall adherence rate were molecule choice and timing of administration. The antibiotic choice was not in accordance with national [24] and international guidelines [8,25,26,27] in almost one out of five of the procedures. This finding is in line with data presented in the literature [12,16,18,28,29]. Choosing an appropriate antibiotic is a critical issue in the correct implementation of SAP. The ideal prophylactic agent should reach a high concentration in surgical site tissue with narrow spectrum of activity to ensure adequate protection. In the present study, broad-spectrum antibiotics, such as cefuroxime and ceftriaxone, were inappropriately used instead of cefazolin. Cefazolin is generally considered the first-choice antibiotic for clean operations, in addition to providing coverage for many clean-contaminated operations. The timing of the administration of the SAP is also disappointing, since in four out of ten procedures, the antibiotic was given at an inappropriate timing, mainly after surgical incision, when it is no longer effective. The results of the multivariate analysis showed that an older age and poor health status of the patients were predictive of having an appropriate SAP timing, whereas an inappropriate timing was more frequent in obstetrics, gynecological, and urological procedures, whereas plastic and reconstructive surgery showed better adherence. Wide variation in SAP appropriateness across surgical procedure groups was also noted in previous studies. In particular, the results of two investigations conducted in Australia and in China showed that the overall appropriateness of SAP was higher than that observed in the present study, ranging from 33.7% for dentoalveolar surgery to 68.9% for neurosurgery and from 13.9% for intestinal surgery to 42.1% for orthopedic surgery, respectively [30,31].

Third, appropriate antibiotic route of administration, duration, and dosage were observed in almost the entire cohort, showing high adherence to guidelines that was higher than that reported in a previous survey conducted in the same geographic area [10]. These figures could be the first outcome of the increasing attention paid to the prudent use of antibiotics. The Italian Ministry of Health issued its first four-year National Action Plan to tackle AR [32] by strengthening and further developing initiatives against AR and HAI. Regulatory frameworks around SAP placed considerable emphasis on improving practices regarding antibiotic use.

Although the study findings shed light on understanding physicians’ practices regarding appropriate implementation of SAP, while interpreting the results, a potential limitation exist. The patients were recruited from seven hospitals located in two geographic areas in Southern Italy and, therefore, the results may not reflect the real situation throughout the country, which limits the generalizability of the study findings.

## 4. Materials and Methods

### 4.1. Study Design and Sampling

The cross-sectional study was performed between May and July 2019. A two-stage cluster sampling procedure was used, and participants were selected from 7 public hospitals in the Campania and Calabria Regions of Italy. First, 7 hospitals were selected randomly. Then, participants were randomly selected from each hospital. Patients aged 18 years and older admitted to surgical wards (general and specialist) that underwent elective minor surgical procedures were eligible for enrollment in this study. A sample size of 480 patients would be required, assuming a 50% prevalence of appropriate use of SAP [10,11,33,34], a 95% confidence interval, a 5% error, and a response rate of 80%.

### 4.2. Procedure and Survey Instrument

Before the study, the research team, through an invitation letter, contacted all medical directors of the selected hospitals. They were informed about the purposes and the methodology of the study and requested permission to conduct it. Additionally, they were assured about the anonymity and confidentiality of the collected data. After approval was granted, two trained interviewers not directly involved in patient care visited each surgical ward of the selected hospitals and approached the eligible patients. The interviewers provided the potential participants information about the study, indicated that the questionnaire completion was voluntary and anonymous, and asked if they wanted to participate. Written informed consent was obtained from each respondent before the interview. No incentives were provided.

Detailed data were collected through the interview regarding demographic and health status characteristics (gender, age, educational level, occupation, comorbidities measured with the Charlson comorbidity index score [35], use of antibiotics in the previous month) and from the medical record after the patient was discharged (ward and length of hospital stay, surgical procedure, wound classification, duration of surgical procedure in minutes, type of anesthesia, implant of prosthesis, American Society of Anesthesiologists (ASA) score for the assessment of the patient’s pre-anesthesia medical comorbidities [36]). The following information on each antibiotic administrated for prophylaxis was also collected: molecule choice, timing, duration, dosages, and route of administration. If the indication of the prescription was not clearly documented in the patient record, the treating surgeon was contacted.

Two trained members of the research team assessed the appropriateness of the SAP for each surgical procedure and each prescription given for the surgery. SAP was deemed to be appropriate if it was not administered without indication or if it was administered in the presence of indication and all evaluated items (drug choice, timing, duration, dosages and route of administration) in accordance with the Italian guidelines of perioperative antibiotic prophylaxis in adults [24] and of the Society of Ophthalmology [37]. The timing of SAP was judged as appropriate if the antibiotic was administered within 1 h prior to surgical incision; in all other cases, it was defined inappropriate except for cataract surgery where topical antibiotics starting the morning after surgery were recommended [37]. There were no differences between hospitals in the protocols used for SAP.

### 4.3. Pilot Study and Ethical Approval

The data collection tool was pretested on a random sample of 25 patients not included in the final sample, and the necessary amendment was made to the final data collection tool. The Ethics Committee of the Teaching Hospital of the University of Campania “Luigi Vanvitelli” approved the study protocol and the data collection form (approval number 201, 27 March 2019).

### 4.4. Statistical Analysis

All analyses were performed using the statistical software Stata version 15 [38]. Firstly, descriptive analysis was performed in order to describe the several characteristics of the patients. Secondly, unadjusted testing for association between the independent variables and the outcomes of interest was done using chi-square and Student’s t-test. All independent variables with a *p*-value equal or less than 0.25 in the univariate analyses were introduced in the multivariate stepwise logistic regression models constructed to identify factors associated with these two outcomes of interest: SAP completely in accordance with the evidence-based guidelines (Model 1) and appropriate timing if the antibiotic was administered within 1 h prior to surgical incision (Model 2). The following independent variables were included in the models: gender (male = 0, female = 1), age (continuous, in years), Charlson comorbidity index score (0 = 0; ≥1 = 1), history of antibiotic allergy (no = 0; yes = 1), use of antibiotics in the previous month (no = 0; yes = 1), type of hospital admission (day surgery = 0; ordinary = 1), surgical ward (general = 0; specialties = 1), surgical procedure groups (abdominal/vascular/breast surgery = 1; ophthalmology = 2; otolaryngology, head and neck/dentoalveolar surgery = 3; obstetrics/gynecological/urological surgery = 4; orthopedic surgery = 5; plastic and reconstructive surgery = 6), surgical wound classification (clean = 0; clean-contaminated/contaminated/dirty-contaminated = 1), type of anesthesia (general = 0; local = 1), treatment involving prosthetic implant (no = 0; yes = 1), and duration of surgical procedure (continuous, in minutes).

The significant level choices for the inclusion and elimination of the variables in the multivariate stepwise logistic regression models were *p*-values of 0.2 and 0.4 respectively. The results of the multivariable models are expressed as odds ratio (ORs) with 95% confidence interval (95% CIs). Two-tailed tests were conducted, and a *p*-value ≤ 0.05 was considered statistically significant.

## 5. Conclusions

In conclusion, the present study, given the vastly limited literature from Italy, provides a snapshot of the current SAP approach in elective minor surgical procedures useful to support and direct AMS interventions, guidelines development, and hospital policy. Indeed, substantial discrepancies between SAP guidelines and practices in elective minor surgery were shown. Antibiotics cannot be indiscriminately administered to any surgical patient in order to prevent SSIs, since SAP is not necessary in many surgical situations. This study underlines that aspects of SAP that need improvement are related to molecule choice, time of administration, and specific surgical procedures. It is necessary that hospital managers involve surgeons and anesthesiologists in initiatives tailored to specific common reasons for inappropriateness to optimize SAP prescribing.

## Figures and Tables

**Table 1:** Demographic, clinical, and admission characteristics of patients according to surgical antibiotic prophylaxis (SAP) approach.

Characteristics	All Procedures (602)		Complete Accordance (271)			Appropriate Timing (287)		
	N (%)	Mean ± SD	N (%)	Mean ± SD	*p*	N (%)	Mean ± SD	*p*
**Gender**					0.366			<0.001
Male	285 (47.4)		123 (45.4)			110 (38.5)		
Female	316 (52.6)		148 (54.6)			176 (61.5)		
**Age, in years**		61.7 ± 18.1		63.6 ± 17.8	0.020		63.1 ± 16.7	0.106
**Prior antibiotic allergies**					0.096			0.247
Yes	35 (5.8)		11 (4.1)			13 (4.5)		
No	567 (94.2)		260 (95.9)			274 (95.5)		
**Type of hospital admission**					<0.001			0.136
Ordinary	374 (62.1)		219 (80.8)			182 (63.4)		
Day surgery	228 (37.9)		52 (19.2)			105 (36.6)		
**Ward of hospital stay**					0.150			0.014
General surgery	164 (27.2)		66 (24.3)			53 (18.5)		
Surgical specialties	438 (72.8)		205 (75.7)			234 (81.5)		
**Surgical procedure groups**					<0.001			0.005
Ophthalmology	270 (44.8)		157 (57.9)			152 (52.9)		
Abdominal, vascular, breast	168 (27.9)		68 (25.1)			53 (18.5)		
Obstetrics, gynecological, urological	65 (10.8)		2 (0.7)			40 (13.9)		
ORL, head and neck, dentoalveolar	57 (9.5)		19 (7.1)			26 (9.1)		
Plastic and reconstructive	32 (5.3)		25 (9.2)			6 (2.1)		
Orthopedic	10 (1.7)		0 (0.0)			10 (3.5)		
**Antibiotics use in the previous month**					0.016			0.101
Yes	62 (10.3)		19 (7.1)			24 (8.4)		
No	540 (89.7)		252 (92.9)			263 (91.6)		
**Charlson Comorbidity index**					0.606			<0.001
0	200 (33.2)		93 (34.3)			103 (35.9)		
≥1	402 (66.8)		178 (65.7)			184 (64.1)		
**Anesthesia**					<0.001			0.619
General	92 (15.8)		12 (4.8)			52 (18.7)		
Local	489 (84.2)		244 (95.2)			225 (81.3)		
**Surgical procedure duration, minutes**		23.4 ± 21.8		18.1 ± 11.7	<0.001		23.8 ±23.7	0.918
ASA score					0.004			0.275
<3	352 (90.7)		83 (98.8)			103 (91.9)		
≥3	36 (9.3)		1 (1.2)			9 (8.1)		
**Surgical wound classification**					<0.001			0.843
Clean	478 (79.4)		245 (90.4)			222 (77.3)		
Other	124 (20.6)		26 (9.6)			65 (22.7)		
**Involving a prosthetic implant**					<0.001			<0.001
Yes	158 (27.1)		4 (1.6)			34 (12.3)		
No	425 (72.9)		253 (98.4)			244 (87.7)		

**Table 2:** SAP administration in procedures with indication according to drug choice, route of administration, timing, duration, and dosage.

Surgical Procedures with SAP Indication and Administration (322)	n (%)
**Drug choice**	
Optimal (concordant with guidelines)	262 (81.4)
Inadequate (unsuitable choice for SAP)	60 (18.6)
**Route of administration**	
Appropriate	307 (95.3)
Inappropriate	15 (4.7)
**Timing**	
Appropriate (within 60 min before incision)	193 (60.1)
Inappropriate (>60 min before incision)	125 (39)
Inappropriate (within 24 h after incision)	3 (0.9)
**Duration**	
Appropriate (within 24 h)	307 (95.3)
Inappropriate (over 24 h)	15 (4.7)
**Dosage**	
Appropriate	308 (95.6)
Inappropriate	14 (4.4)
**Appropriate drug choice, route of administration, timing, duration, and dosage**	166 (51.5)

Number for each item may not add up to total number of surgical procedures due to missing values.

**Table 3:** Multiple logistic regression analysis results examining appropriate antibiotic prophylaxis in surgical procedures and appropriateness of timing of SAP administration according to several explanatory variables.

Variable	OR	SE	95% CI	*p*
**Model 1. Appropriate antibiotic prophylaxis in surgical procedures**				
Log likelihood = −182.3, χ^2^ = 383.3 (7 df), *p* < 0.0001, No. of obs = 546				
Local anesthesia	8.45	4.2	3.13–22.85	<0.001
Treatment not involving prosthetic implant	0.01	0.01	0.01–0.02	<0.001
Ordinary admission	2.93	0.98	1.52–5.66	0.001
Surgical procedure groups				
Abdominal, vascular, breast	1 *			
Ophthalmology	2.56	0.8	1.4–4.72	0.002
Plastic and reconstructive	6.14	4.11	1.65–22.83	0.007
Obstetrics, gynecological, urological	0.14	0.11	0.03–0.65	0.013
Prior antibiotic allergies	0.61	0.35	0.2–1.88	0.394
**Model 2. Appropriate timing of SAP administration in surgical procedures with SAP indication**				
Log likelihood = −240.32, χ^2^ = 165.9 (10 df), *p* < 0.0001, No. of obs = 475				
Treatment not involving prosthetic implant	0.06	0.02	0.03–0.11	<0.001
Charlson comorbidity index =0	0.22	0.09	0.11–0.48	<0.001
Surgical procedure groups				
Abdominal, vascular, breast	1 *			
Obstetrics, gynecological, urological	0.31	0.13	0.13–0.71	0.005
Plastic and reconstructive	20.33	23.5	2.11–195.9	0.009
Ophthalmology	0.64	0.22	0.32–1.27	0.202
ORL, head and neck, dentoalveolar	0.59	0.29	0.23–1.54	0.283
Older	1.03	0.01	1.01–1.05	0.017
Females	1.49	0.35	0.94–2.37	0.092
Not prior antibiotic allergies	0.55	0.26	0.21–1.41	0.212
Not prior antibiotics use	0.64	0.25	0.3–1.39	0.261

* Reference category.
